# Permeation Properties of Water Vapor through Graphene Oxide/Polymer Substrate Composite Membranes

**DOI:** 10.3390/membranes13050533

**Published:** 2023-05-21

**Authors:** Risa Takenaka, Norihiro Moriyama, Hiroki Nagasawa, Masakoto Kanezashi, Toshinori Tsuru

**Affiliations:** Chemical Engineering Program, Graduate School of Advanced Science and Engineering, Hiroshima University, 1-4-1 Kagamiyama, Higashi-Hiroshima 739-8527, Japan

**Keywords:** graphene oxide (GO), dehumidification, porous substrate, filtration method, casting method

## Abstract

Graphene oxide (GO) has attracted attention as an excellent membrane material for water treatment and desalination owing to its high mechanical strength, hydrophilicity, and permeability. In this study, composite membranes were prepared by coating GO on various polymeric porous substrates (polyethersulfone, cellulose ester, and polytetrafluoroethylene) using suction filtration and casting methods. The composite membranes were used for dehumidification, that is, water vapor separation in the gas phase. GO layers were successfully prepared via filtration rather than casting, irrespective of the type of polymeric substrate used. The dehumidification composite membranes with a GO layer thickness of less than 100 nm showed a water permeance greater than 1.0 × 10^−6^ mol/(m^2^ s Pa) and a H_2_O/N_2_ separation factor higher than 10^4^ at 25 °C and 90–100% humidity. The GO composite membranes were fabricated in a reproducible manner and showed stable performance as a function of time. Furthermore, the membranes maintained high permeance and selectivity at 80°C, indicating that it is useful as a water vapor separation membrane.

## 1. Introduction

Water separation, including the desalination of seawater, production of ultrapure water, wastewater treatment, dehydration of compressed air, and dehydration of alcohols, such as methanol and isopropanol, is in high demand and has been realized in both the gaseous and liquid phases [1,2,3,4,5]. Various methods, such as distillation and extraction, are used for the separation and recovery of water in both phases. Membrane technology is a useful energy-saving and compact separation method [6,7]. 

Graphene oxide (GO) is a 2D material with a high aspect ratio, high mechanical strength, a large surface area, and high chemical stability. GO also has many oxygen functional groups, such as carboxyl, hydroxyl, epoxy, and carbonyl groups, at the edge and basal surface of the sheet, which are negatively charged in water [8]. The electrostatic repulsion-induced high dispersibility and hydrophilic characteristics promote stacking in water [9,10]. These excellent properties have made GO an attractive membrane material for water separation [11,12]. Nair et al. [13] found that the GO membranes completely blocked the permeation of small gases, such as He, or vapors, and liquids, such as organic solvents. Notably, only water molecules can permeate GO membranes extremely quickly. This is because in the dry state, the layer spacing, determined by van der Waal forces acting between the GO nanosheets, is insufficient even for He, the smallest molecule, to permeate. However, in the wet state, the spaces between the unoxidized graphene surfaces function as a two-dimensional pore network that allows the frictionless flow of a single layer of water molecules. Sun et al. prepared freestanding GO membranes using the drop-casting method and evaluated the RO permeation properties of sodium salts, heavy metal salts, and organic pollutants [14]. Zhang et al. deposited GO on polydopamine-coated polysulfone substrates using the layer-by-layer method and cross-linked it with 1,3,5-benzene tricarbonyl trichloride (TMC) [15]. The fabricated GO membranes exhibited high rejection rates for organic dyes with molecular weights of approximately 500 Da, and the permeation flux of water exceeded that of most commercially available nanofiltration membranes. In addition to being stable and extremely hydrophilic in a variety of organic solvents, such as acetone and methanol, GO membranes are suitable for the nanofiltration of organic solvents, owing to their layer spacing, which can be precisely controlled [16,17,18]. Huang et al. prepared GO composite membranes on ceramic hollow fiber supports via vacuum aspiration, which exhibited high flux and separation factors in the dehydration of dimethyl carbonate [19]. 

GO membranes can be fabricated via filtration [20,21], spin-coating [22], casting [14], dip-coating [23], layer-by-layer [24], and other methods. Among these, filtration is the most widely used because of its ease of operation. However, there are few reports on the casting method, which mostly focus on the preparation of freestanding membranes. Self-supporting membranes are insufficient for industrial applications because of their low mechanical strength. To address this issue, GO separation layers were formed on porous supports. Zhang et al. fabricated GO composite membranes using ceramic, polyacrylonitrile (PAN), and polycarbonate (PC) as supports and reported that the surface morphology and chemical structure of the substrates and the bulk pore structure affected the adhesion of the GO separation layer to the substrate and the nanofiltration performance [25].

Although GO membranes have been extensively studied for liquid phase separation, such as nanofiltration [24,26], reverse osmosis [27,28], and osmotic vaporization [29,30], only a limited number of papers have been published on their application in water vapor separation. Shin et al. prepared a 6-µm-thick freestanding GO membrane using the casting method. The GO used to fabricate this membrane was synthesized from graphite powder using a modified Hummers method and was applied to water vapor separation for the first time [31]. They reported that the prepared membranes showed excellent performance in both permeance and selectivity, with a water vapor permeance of 1.01 × 10^−5^ mol/(m^2^ s Pa) and a H_2_O/N_2_ higher than 10^3^ at 31.5 °C. Petukhov et al. [32] coated GO on anodic aluminum oxide (AAO) to obtain GO/AAO composite membranes using spin-coating and pressure filtration methods. They used AAO with different pore sizes and thicknesses as supports and applied them to the dehumidification of various gases, at 23–25 °C, such as N_2_, CH_4_, and CO_2_. The composite membranes exhibited high performance comparable to that reported by Shin et al. The mechanical strength was improved using AAO supports. They also reported that a reduction in the edge length of the GO sheet improved the water transport efficiency and gas separation performance. To the best of our knowledge, no other studies have reported the application of GO membranes in dehumidification. Currently, GO-based composite membranes for dehumidification in the gas phase are limited to AAO/GO membranes, and water permeance is evaluated over a limited temperature range, that is, room temperature. As a typical ceramic material, AAOs are hydrophilic and suitable for GO coating, but their brittleness makes the scale-up difficult. To clarify the possibility of dehumidification using GO-based membranes from a practical viewpoint, the fabrication of GO membranes, including the coating method, coating amount, and type of substrate, must be optimized, and the permeation properties of water vapor should be evaluated with considerations on the temperature, humidity, and gas.

In this study, GO–polymer composite membranes were fabricated on three different porous polymeric substrates, namely polyethersulfone (PES), cellulose ester (CE), and polytetrafluoroethylene (PTFE), via suction filtration and the casting method. The membrane fabrication method and coating amount were investigated by evaluating the water vapor permeation properties, such as the dependency of water permeance and selectivity over a wide range of humidities and temperatures, to achieve a practical level of membrane performance. 

## 2. Experimental

### 2.1. Material

The aqueous GO dispersion (4 mg/mL) was purchased from Graphenea, Inc. (San Sebastián, Spain). The microporous membranes that were used as substrates were PES (pore size: 0.22 µm, Sigma-Aldrich, Tokyo, Japan), CE (pore size: 0.1 µm, Advantech, Tokyo, Japan), and PTFE (pore size: 0.1 µm, Advantech, Tokyo, Japan) with a diameter of 25 mm. The properties of the substrates used in this study are summarized in Table S1 of the Supplementary Materials. The GO coating solutions were adjusted to a concentration of 0.01–3.9 mg/cm^2^ and a pH of 2.2–2.8 by adding water as a solvent to a predetermined amount of GO aqueous dispersion. The solution was sonicated at 28 kHz for 10 min prior to coating.

### 2.2. Membrane Fabrication

GO-coated membranes are fabricated by suction filtration or casting. In the suction filtration method, water is first used to wet the PES and CE substrates, and ethanol is used to wet the pores of the PTFE substrate. An adjusted amount of solution was then poured and filtered using a suction pump. In the casting method, the coating solution was cast directly onto the substrate with a gap of 150 µm between the glass plate and the glass rod. The lower the GO concentration, the more difficult it is to obtain a uniform coating layer. The coated substrates were dried overnight at room temperature to obtain the GO membranes. Using suction filtration, the amount of coating was calculated from the filtration volume, GO concentration, and membrane area. The mass difference between the membranes before and after casting, the GO concentration, and the film area were used in the casting method. Further details can be found in Figure S1 of the Supplementary Materials.

### 2.3. Characterization

The X-ray diffraction (XRD) patterns of the GO membranes were obtained using an X-ray diffractometer (D2 PHASER, BRUKER, Kanagawa, Japan) equipped with an X-ray generator operating at 30 kV and 10 mA. Data were collected in the range of 2 θ (5.0−80.0°) at an angular resolution of 0.048°/s. The morphology and structure of the GO membrane surface and cross-section were observed using field emission scanning electron microscopy (FE-SEM; Hitachi S-4800, Tokyo, Japan) at accelerating voltages ranging from 3 to 10 kV. The samples were dried naturally overnight, and cross-sections were prepared by immersing the membranes in liquid nitrogen and sectioning them with a feather cutter. Water vapor adsorption was measured using a BELSORP-max analyzer (BELL Corp., Osaka, Japan). Figures S2–S4 in the Supplementary Materials summarize the structural and chemical characterizations of commercially available GO dispersions, including dynamic light scattering analysis (DLS, Malvern Nano ZS, Tokyo, Japan), transmission electron microscopy (TEM, JEOL2010 Ltd., Tokyo, Japan), thermogravimetric analysis (TG, TG-50, Shimazu, Kyoto, Japan), and Fourier transform infrared spectroscopy (FT-IR, FT/IR-4100, JASCO, Tokyo, Japan).

### 2.4. Membrane Performance Measurements

**Figure 1:**
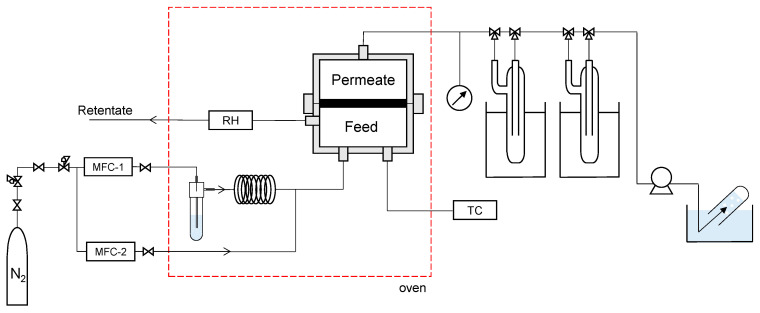
Schematic of vapor permeation apparatus. (MFC: mass flow controller, RH: hygrometer, TC: temperature controller).

Vapor permeation (VP) measurements were conducted using the apparatus shown in Figure 1. Ultrapure water was half-filled into the bubbler, and water vapor was supplied by bubbling nitrogen at a flow rate of 1 L/min. It was confirmed that the feed flow rate was large enough for the membrane area (2 cm^2^) and permeance of water vapor, approximately up to 10^−5^ mol/(m^2^ s Pa). Hence, it can be assumed that the composition of the retentate and feed is insignificant. The fabricated membrane was fixed to the module with an O-ring and placed in an oven, where the temperature was maintained at a predetermined level. The feed stream was maintained at atmospheric pressure (101.3 kPa), and the permeate stream was evacuated at a pressure of less than 1.0 kPa. The permeated water vapor was collected in a cold trap using liquid nitrogen, whereas the permeated nitrogen was collected via water displacement from the outlet of the vacuum pump. It should be noted that the detection limit of nitrogen permeance for each experiment was estimated, assuming that 0.1 cc of N_2_ was collected for 20 min at a nitrogen partial pressure difference of 1 bar between the feed and permeate. The oven temperature was controlled to reach a predetermined temperature in the range of 25−80 °C. Humidity was controlled at 0−100% by adjusting the mass flow rate of nitrogen through two lines for dry and wet gases (MFC-1 and -2, respectively). The relative humidity (RH) was monitored using a HYGROFLEX 5-SERIES instrument (Rotronic, Tokyo, Japan). 

The permeation flux (*J_w,i_* [kg/(m^2^ h)]) and permeance (*P_i_* [mol/(m^2^ s Pa)]) of each component, *i*, were calculated using the following equations:

(1) $$J_{w,i}=\frac{M_{i}}{At}=M_{w}J_{i}$$

(2) $$P_{i}=\frac{J_{i}}{∆p_{i}}=\frac{J_{i}}{p_{if}-p_{ip}}≅\frac{J_{i}}{p_{if}}$$

In Equation (1), *M_i_* is the mass of the *i*-component, *t* is the collection time, and *A* is the effective membrane area. In Equation (2), *J_i_* is the permeate molar flux of the *i*-component, and Δ*p_i_* is the partial pressure difference between the membrane feed (*p_if_*) and the permeate (*p_ip_*) of the *i*-component, which can be approximated as *p_if_*, owing to the low total pressure of the permeate stream_._

## 3. Results and Discussion

### 3.1. Fabrication Conditions and Water Vapor Permeation Properties of the GO Membrane

**Figure 2:**
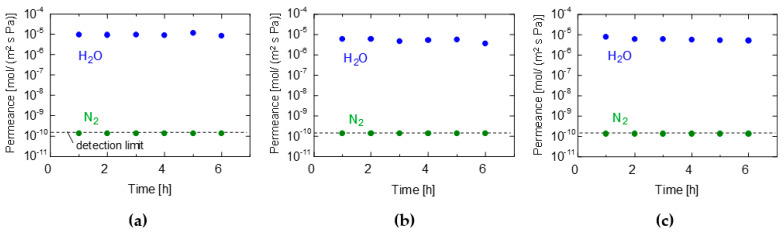
Time courses for the VP performance of (**a**) GO/PES, (**b**) GO/CE, and (**c**) GO/PTFE membranes. (GO coating amount: 0.0565 mg/cm^2^, filtration method, temperature: 25 °C, RH: 90−100%).

Figure 2a–c show the time courses of the water and nitrogen permeance of the GO/PES, GO/CE, and GO/PTFE membranes, respectively. The membranes were prepared using the suction filtration method with 0.0565 mg/cm^2^ of GO coating. The measurement conditions were maintained at 90–100% relative humidity (RH) at room temperature (~25 °C). The water and nitrogen permeances remained almost constant for up to 6 h from the start of the experiment, although the membranes were maintained overnight at room temperature before their use. This indicates that the membranes were stable for at least 6 h, and a steady state was reached in a short time, owing to the thin film thickness. Interestingly, the water permeance was extremely high (~10^−5^ mol/ (m^2^ s Pa)), whereas the nitrogen permeance was below the detection limit (1.0 × 10^−10^). Hence, a high permeance and permeance ratio greater than 10^4^ was obtained. In subsequent studies, when the permeance reached a steady state within a short time, the performances measured 1 h after the beginning of the permeation experiment were considered steady-state values. 

**Figure 3:**
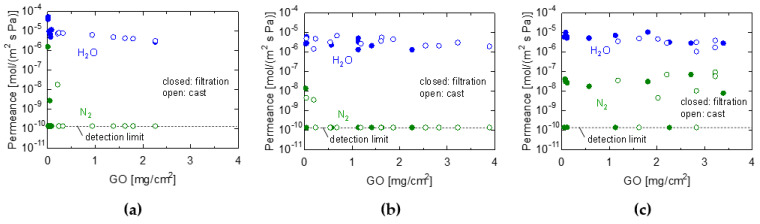
Water and N_2_ permeance of GO membranes prepared by filtration (closed keys) and casting (open keys) with different loading amounts. (**a**) GO/PES, (**b**) GO/CE, and (**c**) GO/PTFE membranes. (Temperature: 25 °C, RH: 90−100%).

Figure 3a–c show the dependence of water permeance on the amount of GO coating in the VP experiment for the PES/GO, CE/GO, and PTFE/GO membranes, respectively. The membranes were fabricated using suction filtration and casting. It should be noted that each plot point for water permeance represents a different membrane, indicating the reasonable reproducibility of membrane fabrication. Therefore, it was confirmed that GO membranes with high permeance and selectivity can be fabricated using these methods. The GO membranes fabricated using the three types of substrates exhibited a separation factor greater than 10^4^ and water permeance larger than 1.0 × 10^−6^ mol/ (m^2^ s Pa). However, the performance of the GO/PTFE membranes varied; some membranes showed approximately the same performance as the GO/PES and GO/CE membranes (H_2_O permeance > 10^−6^ mol/ (m^2^ s Pa), H_2_O/N_2_ > 10^3^), whereas others allowed a N_2_ permeation of 10^−8^−10^−7^ mol/ (m^2^ s Pa). This can be attributed to difficulties associated with coating the PTFE membrane uniformly, owing to its hydrophobicity and its permeance varying widely. However, we clarified that hydrophilic GOs could be successfully coated on porous PTFE substrates, which are typically hydrophobic materials, by pre-filling the PTFE pores with ethanol before coating them. The water permeance gradually decreased as the GO content increased. This was attributed to the increased permeation resistance of the GO separation layer with an increasing membrane thickness, which made it more difficult for the water vapor to permeate [33]. In the suction filtration, the nitrogen permeance was less than ~10^−10^ mol/(m^2^ s Pa) for GO coating amounts greater than 0.01 mg/cm^2^ for PES and 0.03 mg/cm^2^ for CE, whereas those of lower GO coating amounts increased abruptly. In the low coating amount region (~0.03 mg/cm^2^), the water vapor permeance reached as high as ~10^−5^ mol/(m^2^ s Pa). In contrast, in the casting method, a GO coating amount greater than 0.04 mg/cm^2^ is required to fabricate water-selective membranes with a nitrogen permeance of less than ~10^−10^ mol/(m^2^ s Pa). These results indicate that the filtration method used for membrane fabrication can produce thinner membranes with a high-water selectivity. This may be because the casting method requires a higher viscosity in the coating solution used for membrane fabrication, and the concentration of the coating solution must be higher than that in the suction filtration method.

**Figure 4:**
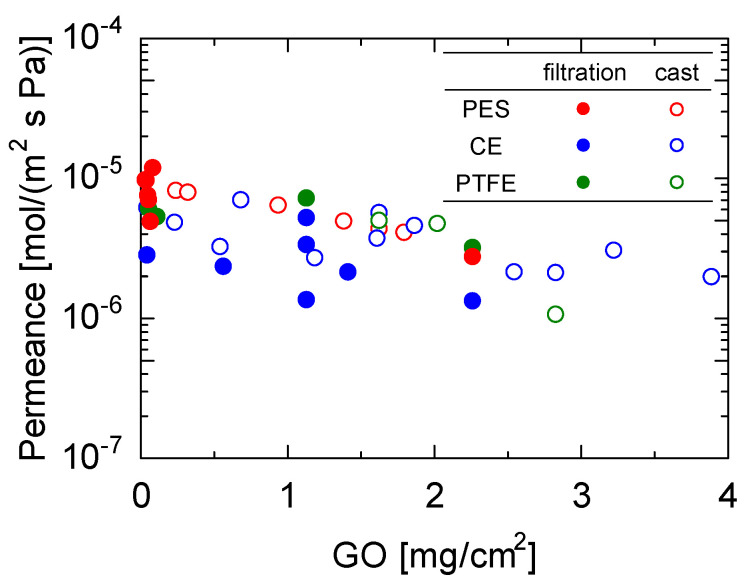
Water permeance of GO membranes prepared by filtration (closed keys) and cast (open keys) with different coating amounts. (Temperature: 25 °C, RH: 90−100%).

Figure 4 shows the water permeance as a function of the amount of GO coating for membranes with H_2_O/N_2_ permeance ratios greater than 10^4^, which corresponds to a N_2_ permeance smaller than 10^−10^ mol/(m^2^ s Pa) (the detection limit). Detailed figures are provided in Figure S5 of the Supplementary Materials. It should be noted that all the data were obtained from Figure 3a–c. PES substrates with the highest porosity exhibited the highest water permeance (1.18 × 10^−5^ mol/ (m^2^ s Pa)) at a GO coating amount of 0.0396 g/cm^2^. Notably, the water permeance of the membranes formed by the casting approach was higher than that formed by the filtration method for GO membranes with similar coating quantities, although the trends were somewhat scattered. This was particularly noticeable for the CE substrates, which have small surface porosities. This will be further discussed based on the XRD measurements later.

**Figure 5:**
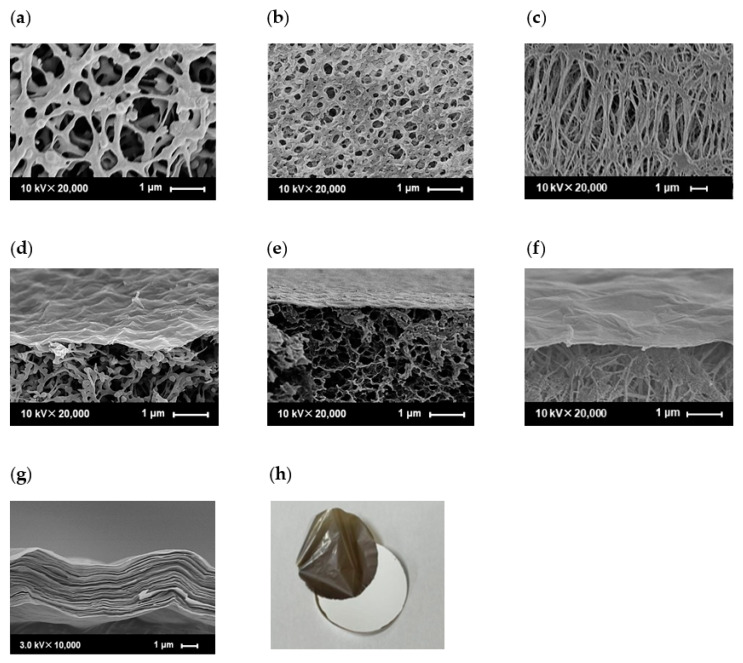
SEM images of different substrates (PES (**a**), CE (**b**), PTFE (**c**)) and cross-sectional morphologies of GO composite membranes ((**d**–**f**)) on different substrates. (GO/PES (**d**), GO/CE (**e**), and GO/PTFE (**f**) were fabricated using the filtration method with GO-coated amounts of 0.0565 mg/cm^2^. The GO layer (**g**) was detached from the PES substrate (**h**) when 0.9394 mg/cm^2^ of GO was coated on the PES substrate using the casting method).

Figure 5 shows cross-sectional FE-SEM images of the PES, CE, and PTFE substrate surfaces and GO membranes fabricated using the filtration and casting methods. Figure 5a–c show that the three substrates had different structures in terms of pore size and porosity. The PES surface pore size ranges from 0.2 to 1.5 µm, the CE surface pore size ranges from 0.1 to 0.5 µm, and the PTFE surface pore size ranges from 0.1 to 2.0 µm, which is different from the nominal pore sizes shown in Table S1. The surface pore sizes and porosities of the PES substrates were larger than those of the CE and PTFE substrates. The cross-sectional images in Figure 5d–f show that thin GO layers with thicknesses of less than 100 nm could be formed on various porous polymer substrates, despite their different surface pore sizes. The GO sheets did not penetrate the pores of the PES, CE, and PTFE. Interestingly, the GO layer covered their surfaces completely, although the surfaces of the substrates with different pore shapes were rough. This can be attributed to the high aspect ratio, with a size of a few micrometers, and the flexibility of the GO. In contrast, the GO membrane (1.63 mg/cm^2^) fabricated using the casting method showed a GO layer thickness of 5 µm, which is significantly thicker than that fabricated using the filtration method, and a structure with many GO sheet-forming layers was observed, as shown in Figure 5g. The casting method does not require suction filtration and is relatively simple; however, the coating solution requires a certain degree of viscosity for the uniform formation of GO layers. Therefore, it was important to increase the GO concentration compared with that used in the filtration method, which is believed to be the cause of the thicker film. Furthermore, delamination between the substrate and the GO layer was observed at high GO contents in the case of the cast membrane (Figure 5h). Notably, no covalent bonds were formed between the GO layers and the substrates because filtration or casting was simply applied to the GO coating. Therefore, the adhesion between the GO layers became stronger than that between the substrate and the GO layers as the amount of the GO coating increased. In contrast, when low amounts of GO coating were used to fabricate the GO membranes using the filtration method, the membranes strongly adhered to the substrate and did not peel during the permeation experiment. 

**Figure 6:**
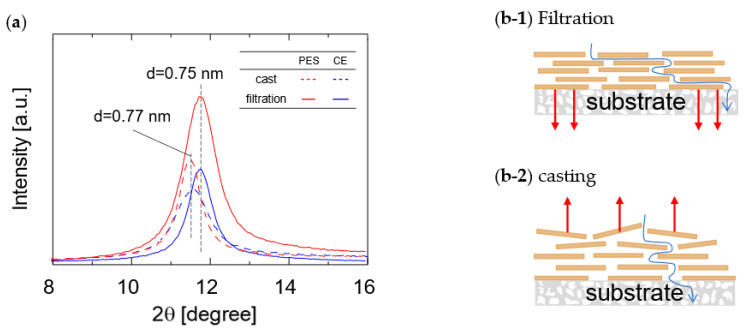
XRD patterns of GO membranes fabricated via filtration and casting methods (**a**) and schematic structures of GO layers prepared via filtration (**b**-**1**) and casting (**b**-**2**).

The GO layer spacing was measured using XRD to investigate the effect of the membrane fabrication method on water vapor permeation properties. Membranes prepared via casting and filtration were dried at room temperature for 20 h before the XRD measurements. Figure 6 shows the XRD patterns of the GO/PES and GO/CE membranes. The XRD patterns of the cast membranes prepared on both the PES and CE substrates showed a broad diffraction peak at approximately 2 θ = 11.5°, which corresponds to the GO layer spacing (d = 0.77 nm) as determined by Sherr’s equation. In contrast, the XRD patterns of the filtrated membranes showed a diffraction peak of around 2 θ = 11.8° for both the PES and CE substrates, which corresponds to a layer spacing of 0.75 nm. The XRD analysis indicated that the layer spacing of the GO membranes prepared on both the PES and CE substrates via the filtration method was narrower than that of those fabricated via the casting method. These data are consistent with those reported by Tsou et al. [34]. During the filtration process, the downward force generated by the decompression during membrane fabrication is assumed to be the reason for the denser structure. As shown in Figure 4, the water permeance of the cast membranes tended to be higher than that of the filtered membranes. This is because water molecules experience greater resistance to permeation through the GO layer when there is less space between the layers. 

### 3.2. Permeation Properties of Water Vapor

**Figure 7:**
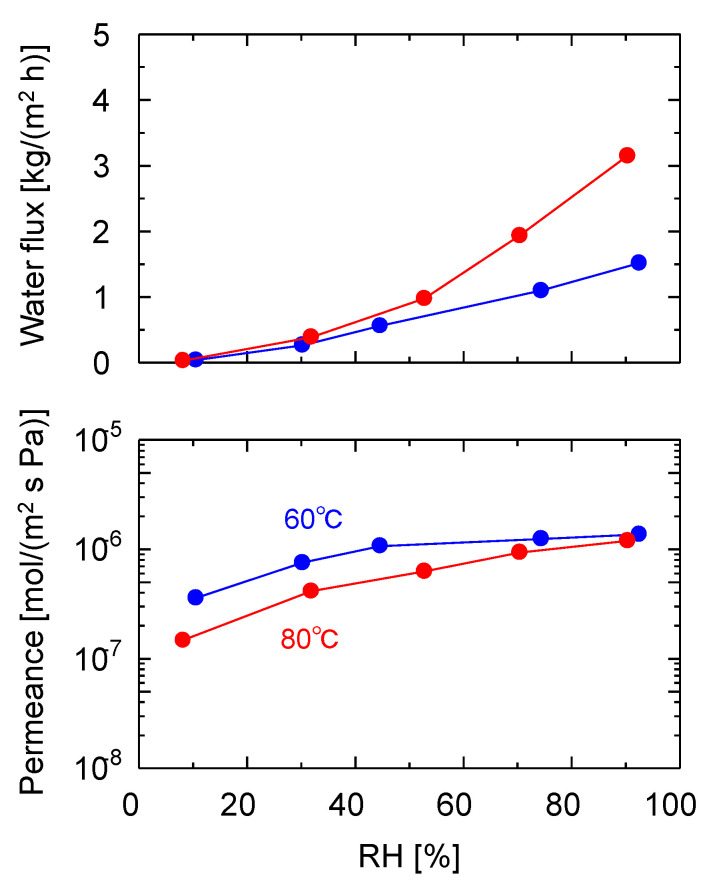
Humidity dependencies of the VP performance of the GO/PES membrane at 60 and 80 °C. (Filtration, GO coating amount: 0. 74 mg/cm^2^).

To further elucidate the water vapor permeation characteristics of the GO membranes, VP experiments using the GO/PES membranes, which showed the highest water vapor permeance among the three substrates, were conducted over a wide humidity range, as shown in Figure 7. The RH was maintained at 60 and 80 °C by adjusting the dry and wet flow rates, and it was monitored using a hygrometer. As shown in the time course of the measurement in Figure S6 in the Supplementary Materials, the measurements started with dry N_2_, and no N_2_ leakage was observed for 1 h. After 1 h, the humidity was increased, step-by-step, every 1–2 h to 90% and then decreased back to 10% and dry N_2_. As the RH increased from 10 to 90% at 80 °C, the water flux increased from 0.0538 to 5.25 kg/(m^2^ h), and the water permeance also increased from 1.83 × 10^−7^ to 2.0 × 10^−6^ mol/(m^2^ s Pa). However, the nitrogen permeate flow rate was below the detection limit at all of the humidities without leakage, indicating that the GO layer was tightly adhered to the substrate and impeded N_2_ permeation. The water permeance at an RH of 10% (from 1050 to 1300 min in Figure S6) was stable, with a similar value as that at the beginning of the measurement (from 190 to 310 min), confirming the high stability of the membrane and reproducible measurements. A similar experiment was performed at 60 °C using the same membrane, and a comparison of the water flux and permeance is shown in Figure 7. A higher flux was observed at 80 °C than at 60 °C because the saturated water vapor pressure at 80 °C (47.3 kPa) was higher than that at 60 °C (19.9 kPa). Hence, it can be deduced that flux is highly dependent on the temperature and humidity.

**Figure 8:**
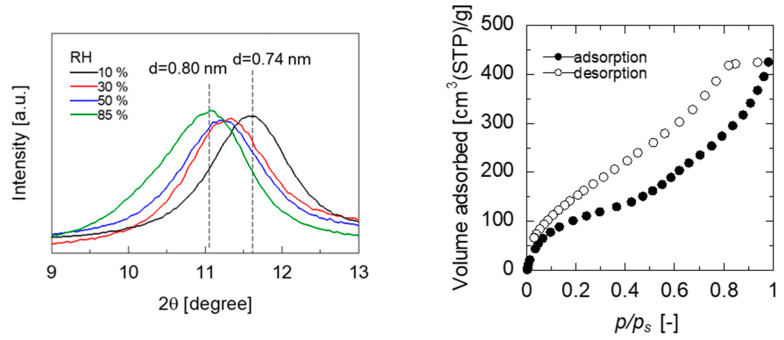
XRD patterns of GO/PES membranes exposed in various RH water vapor (**left**) and water adsorption isotherms at 298 K for a freestanding GO membrane (**right**).

The water permeance increased as the humidity increased, in a manner similar to the surface diffusion, where the adsorbed water diffused along the adsorption gradient. This RH dependence is discussed further based on the difference in the GO layer spacing, as shown in Figure 8. 

XRD measurements of the GO/PES membranes maintained at different humidities were performed to investigate the humidity dependency of the water permeance, as shown in Figure 8. Saturated salt solutions of lithium chloride (RH 10%), magnesium chloride (RH 30%), magnesium nitrate (RH 50%), and potassium chloride (RH 85%) were used to maintain the humidity in the chamber at an arbitrary level. The XRD pattern measured immediately after holding the chamber at 10% humidity for more than 12 h showed a broad diffraction peak originating from the layer spacing of the GO membrane at approximately 2 θ = 11.9° (d = 0.74 nm). In contrast, the XRD pattern measured after keeping the film in the chamber at 90% humidity for more than 12 h showed a diffraction peak originating from the GO membrane layer spacing at approximately 2 θ = 11.0° (d = 0.80 nm). The GO layer spacing increased with the increasing humidity, which is thought to increase water permeance owing to its low resistance to water permeation. Figure 8 also shows the water vapor adsorption isotherms of the GO membranes. A freestanding GO membrane was used for the water adsorption measurement at 25 °C after being evacuated at 80 °C for 10 min to remove the adsorbed water. This mild pretreatment was applied to avoid a reduction of the GOs [35]. Water adsorption on the GO exhibited a type II isotherm, in which the amount of water adsorbed by the GO increased with the increasing humidity. The adsorbed water enlarged the GO layer spacing, which contributed to an increase in water permeance. 

**Figure 9:**
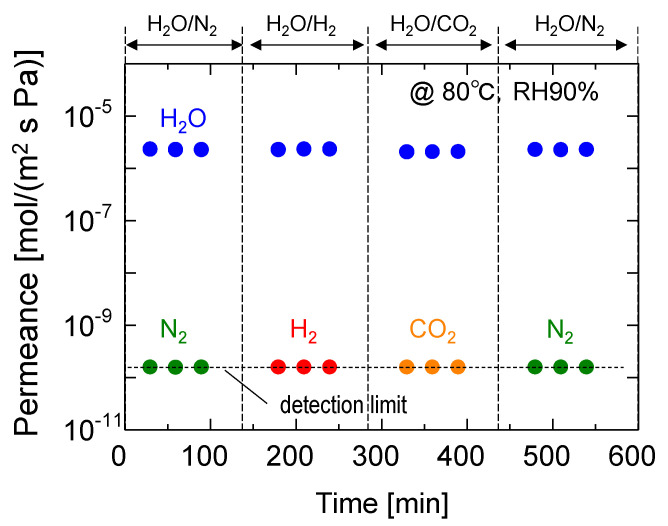
Time course for the separation performance of binary mixtures (H_2_O/N_2_, H_2_O/H_2_, and H_2_O/CO_2_) at 80 °C and an RH of 90%.

Figure 9 shows the time course of the permeance of water vapor and non-condensable binary mixtures, including H_2_O/N_2_, H_2_O/H_2_, and H_2_O/CO_2_. The water and nitrogen permeances measured in the initial and final stages were almost identical, which confirms the reversibility of the membrane performance. Interestingly, the permeation of non-condensable gases, including H_2_, which is the smallest gas, was completely inhibited, and the permeance of each gas was below the detection limit (less than 10^−10^ mol/(m^2^ s Pa)). Notably, the water permeance was unaffected by this type of non-condensable gas, indicating that GO composite membranes are effective for the dehumidification of various gases.

### 3.3. Comparison of Dehumidification Performance

Table 1 compares the water permeance in this study with previously reported dehumidification membranes measured at similar temperatures and humidities. The GO membrane fabricated in this study exhibited higher water permeance than other polymeric membranes. This may be because GO has a sheet-like structure that can be easily stacked, which allows the fabrication of membranes thinner than conventional membranes. Except for the freestanding membranes, the GO membrane was the thinnest. In addition, the high hydrophilicity and excellent water transport properties of the GO layers may contribute to their high water permeance. These results suggest that the GO membranes are promising for water recovery. 

Table 2 summarizes the water permeance and H_2_O/N_2_ separation factors of the water vapor separation system with the GOs as the separation layer. Freestanding GO membranes were first used for dehumidification [31], followed by GO composite membranes coated onto amorphous Al_2_O_3_ porous substrates [32]. In this study, we successfully fabricated GO membranes on different polymeric substrates and evaluated their dehumidification performances. The GO membranes fabricated in this study using a suction filtration were very thin, with a GO layer thickness of less than 100 nm, and they showed high permeance (10^−6^−10^−5^ mol/(m^2^ s Pa) and high selectivity (larger than 1000), similar to those of previously reported GO membranes [31,32]. As discussed previously, water is believed to permeate through the gaps between GO layers, and the permeation resistance is dominated by the gaps between the GO layers. The high water permeance of GO membranes over a wide range of membrane thicknesses can be attributed to the low resistance of the GO layers, confirming GO as a promising material for dehumidification membranes.

## 4. Conclusions

GO membranes were fabricated on inexpensive polymer substrates composed of PES, CE, and PTFE using suction filtration and casting. The prepared GO membranes had a thickness of less than 100 nm for all of the substrates. The membranes exhibited stable and reproducible permeance over a wide range of temperatures and humidity levels. The GO membranes exhibited excellent permeance and selectivity, with a water permeance greater than 1.0 × 10^−6^ mol/ (m^2^ s Pa) and a separation factor larger than 10^4^ at 25 °C and 90−100% RH. The GO membranes fabricated using the casting method showed higher water permeance than those fabricated using suction filtration. This could be attributed to the size of the GO sheet layer spacings, as measured by XRD. The GO membranes also maintained high water permeance and selectivity at 80 °C. The water permeance increased as the layer spacing of the GO sheet increased, and the amount of water adsorbed increased with increasing humidity. This was confirmed by XRD and water vapor adsorption measurements.

## Figures and Tables

**Table 1:** Water vapor permeance of polymeric membranes and GO membranes.

Membrane	Configuration	Thickness [µm]	Temperature [°C]	Feed-in Gas RH [%]	Water Vapor Permeance [mol/ (m^2^ s Pa)]	Ref.
Nafion115^®^ (perfluorosulfonic acid polymer)	Flat sheet	127	30	80	2.6 × 10^−6^	[36]
Nafion115^®^ (perfluorosulfonic acid polymer)	Flat sheet	127	30	100	1.2 × 10^−6^	[37]
Nafion115^®^ (perfluorosulfonic acid polymer)	Flat sheet	127	30	80	5.1−6.0 × 10^−6^	[38]
Polyimide^®^ (perfluorosulfonic acid polymer)	Hollow fiber		40	100	3.3 × 10^−7^	[39]
Polyimide	Hollow fiber		35	30	1.4 × 10^−6^	[40]
Polyetherimide					1.2 × 10^−6^	
Polysulfone					1.1 × 10^−6^	
Pebax^®^ (polyether–polyamide copolymer)	Flat sheet (without support layer)	2	21	40	2.0 × 10^−6^	[41]
	Flat sheet (with support layer)	252			6.7 × 10^−7^	
Sunsep^®^ (perfluorosulfonic acid polymer)	Capillary	250	30	90	7.2 × 10^−6^	[42]
GO	Flat sheet	<0.1	25	90−100	1.18 × 10^−5^	This study

**Table 2:** Water vapor permeance and H_2_O/N_2_ selectivity of GO membranes.

Material of GO	Substrate			Membrane Preparation		GO Thickness [nm]	Water Vapor Permeance * [mol/ (m^2^ s Pa)]	H_2_O/N_2_ Selectivity [–]	Ref.
	Material	Pore Size [µm]		Method	Concentration [mg/mL]				
Modified Hummer’s method	Freestanding	-		Casting	8.9	6000	1.01 × 10^−5^	>10^4^	[31]
Modified Hummer’s method	Anodic aluminum oxide (AAO)	0.01−0.1		Spin-coating	0.5–6.0	730	1.24 × 10^−5^	13,000	[32]
Purchased from Graphenea	Polyethersulfone (PES)	0.22		Filtration	0.1–4	100−3500	2.7 × 10^−6^−1.2 × 10^−5^	>10^4^	This study
	Cellulose ester (CE)	0.1		or		100−5000	1.3–6.9 × 10^−6^	>10^4^	
	Polytetrafluoroethylene (PTFE)	0.1		Casting	0.1–25	100−5000	1.0 × 10^−6^−1.1 × 10^−5^	>10^4^	

* Measurement conditions: 22.5–30.8 °C for [31], 23–25 °C and 80% for [32], and 25 °C and 90% in this study.

## Data Availability

The data presented in this study are available on request from the corresponding author.

## Supplementary Materials

The following supporting information can be downloaded at: https://www.mdpi.com/article/10.3390/membranes13050533/s1, Figure S1: Schematic diagrams of fabrication methods of the GO membrane: (a) casting, (b): filtration; Figure S2: Particle size distribution of GO sols (left) and TEM image of GO (right); Figure S3: TG curve of GO in N_2_ flow (50 cc/min) at a heating rate of 10 °C/min; Figure S4: FT-IR spectra of GO film coated on KBr plates; Figure S5: Water permeance as a function of GO coating amounts for membranes prepared by filtration (a) and cast (b), and membranes of coating amounts less than 0.15 mg/cm^2^ (c). (Temperature: 25 °C, RH: 90–100%); Figure S6: Time course of VP performance at different humidity levels (0–90%) through the GO/PES membrane at 60 and 80 °C. (Filtration, GO coating amount: 0.074 mg/cm^2^); Table S1: Properties of substrates used in this study.
