# Supplementary material for: Permeation Properties of Water Vapor through Graphene Oxide/Polymer Substrate Composite Membranes
Source: Membranes (Basel). 2023 May 21;13(5):533. doi: 10.3390/membranes13050533 (PMC10222251; doi:10.3390/membranes13050533)
Supplement: Supplementary file 1 [file membranes-13-00533-s001.zip › membranes-2381491-supplementary.pdf]

Support information

Preparation of Graphene Oxide/Polymer Substrate Composite Membranes and Evaluation of Water Vapor Permeation Properties

Risa Takenaka, Norihiro Moriyama, Hiroki Nagasawa, Masakoto Kanezashi, Toshinori Tsuru *

Chemical Engineering Program, Graduate School of Advanced Science and Engineering, Hiroshima University, 1-4-1 Kagamiyama, Higashi-Hiroshima 739-8527, Japan
*Corresponding author: E-mail address: tsuru@hiroshima-u.ac.jp

---------SI-1. Properties of substrates------------

Table S1. Properties of substrates used in this study.

**Substrate**	**Pore size (nominal)*1 [µm]**	**Porosity*1 [%]**	**N_2 permeance*2 [mol/ (m^2 s Pa)]**
Polyethersulfone (PES)	0.22	83.2	7.6×10^{-5}
Cellulose ester (CE)	0.1	42.3	2.0×10^{-5}
Polytetrafluoroethylene (PTFE)	0.1	67.3	6.5×10^{-5}

*1) from product data sheet *2) measured at 25 °C, Feed: 100 kPa, permeate < 1 kPa

---------SI-2. Membrane preparation------------

Figure S1 schematically shows how GO membranes were fabricated using casting (a) and filtration (b). In the suction filtration method, the coating amount per unit, A [mg/cm^2], was calculated from the filtration volume, V [cm^3], GO concentration, C_{GO} [mg/cm^3], and membrane area, S [cm^2], as follows:

$$A = \frac{VC_{GO}}{S}$$

For the casting method, the mass difference between the membranes before and after casting, ΔM [g], GO concentration, C_{GO} [mg/g], and membrane area, S [cm^2], were used to obtain the coating amount per unit: A [mg/cm^2]. The density of the GO solution was assumed to be 1 g/cm^3.

$$A = \frac{\Delta MC_{GO}}{S}$$

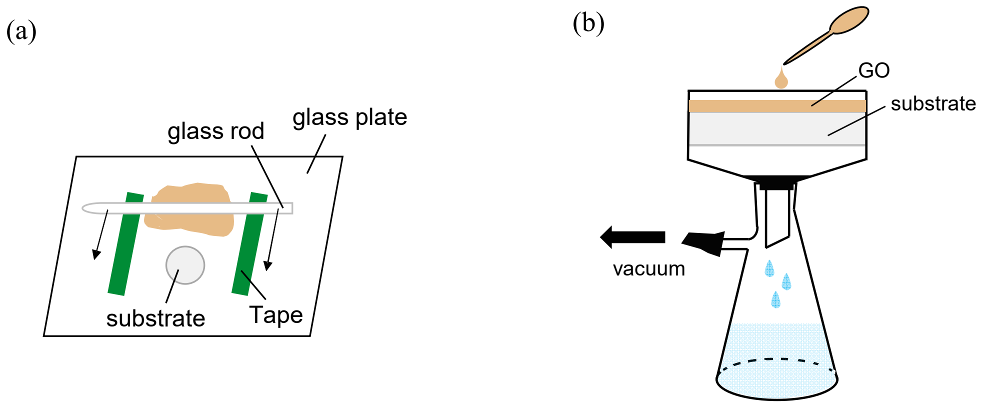

Figure S1. Schematic diagrams of fabrication methods of the GO membrane: (a) casting, (b): filtration

---------SI-3. Characterization of GO particle sizes in liquid and solid phases------------

The particle size of the GO solutions after dilution (0.1 wt %) were measured via dynamic light scattering (DLS, Malvern Nano ZS). The morphology of GO was confirmed using transmission electron microscopy (TEM JEOL2010 Ltd., Japan). Both analyses showed that the GO particles were several micrometers in sheet size, which was sufficiently large for the formation of GO layers on microfiltration membranes with pore sizes of 0.1 - 0.2 µm.

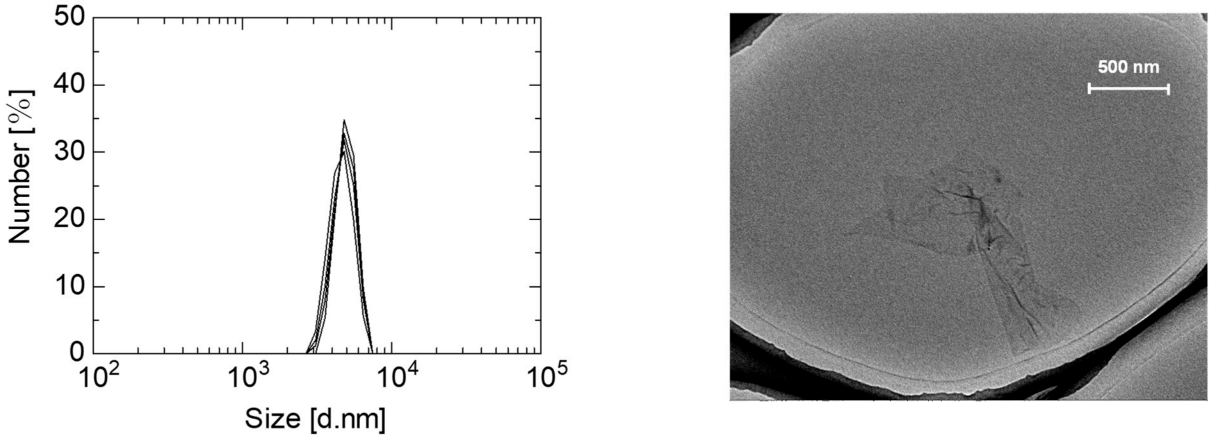

Figure S2. Particle size distribution of GO sols (left) and TEM image of GO (right).

---------SI-4. Characterization of chemical structures of GOs------------

Thermogravimetric analysis (TGA) was conducted using TG-50 (Shimazu, Japan) under nitrogen flow (50 cc/min) after maintaining the GO samples at 100 °C, followed by a constant heating rate of 10 °C/min. The

weight loss occurs in two steps: the first weight loss at 200-250°C and a gradual decrease at 250-500°C, which can be ascribed to the removal of oxygen-containing functional groups from the GO surface and the decomposition of the remaining oxygen-containing functional groups and the carbon skeleton of GO.

GO films coated on KBr plates were evaluated via Fourier-transform infrared spectroscopy (FTIR-4100, JASCO, Japan) over a wavenumber ranging from 500 to 4,000 cm^{-1}. The broad peak centered at approximately 3400-3500 cm^{-1} corresponds to the stretching vibrations of the hydroxyl (O-H) groups. Meanwhile, C=O and C-O stretching vibrations were detected at approximately 1720-1730 cm^{-1} and 1050 cm^{-1}, which correspond to carbonyl (C=O) groups in the carboxylic acid functional groups and ethers (C-O-C) on the surface of GO. Notably, a peak corresponding to the stretching vibrations of C=C bonds was observed at approximately 1600-1650 cm^{-1}.

Both the TG curve (Figure S3) and FTIR spectra (Figure S4) were similar to those of typical GOs prepared using the Hummers' method.

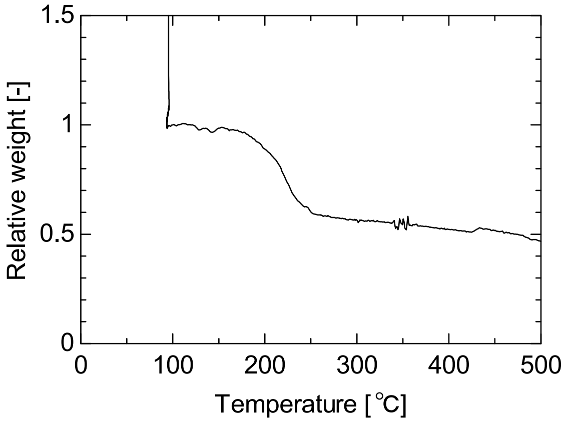

Figure S3. TG curve of GO in N_2 flow (50 cc/min) at a heating rate of 10 °C/min.

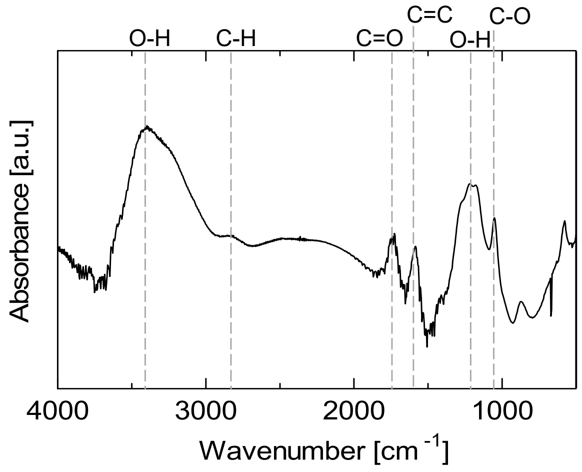

Figure S4. FT-IR spectra of GO film coated on KBr plates.

---------SI-5. Water permeance of GO composite membranes of different GO coating amounts ------------

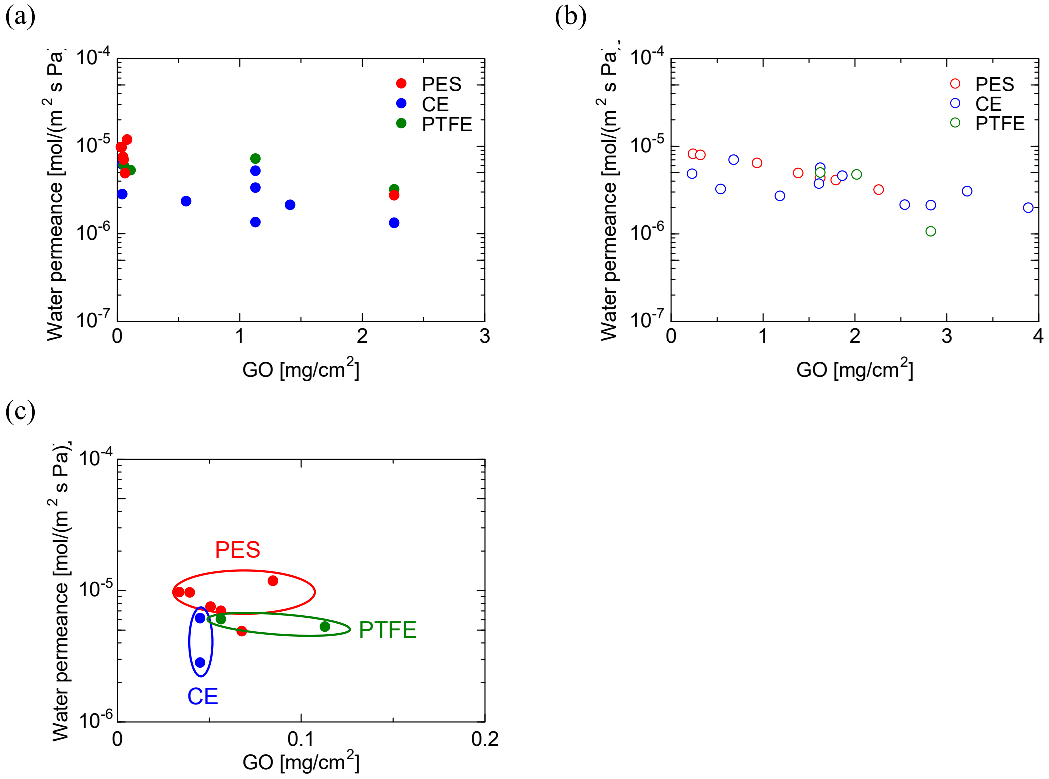

Figure S5. Water permeance as a function of GO coating amounts for membranes prepared by filtration (a) and cast (b), and membranes of coating amounts less than 0.15 mg/cm^2 (c). (Temperature: 25 °C, RH: 90-100%)

--------SI-6. Time course of permeances during the measurement of humidity dependency ------------

Figure S6 shows the time course of VP performance of H_2O/N_2 binary mixtures in a wide range of relative humidity (RH) at 60 and 80 °C.

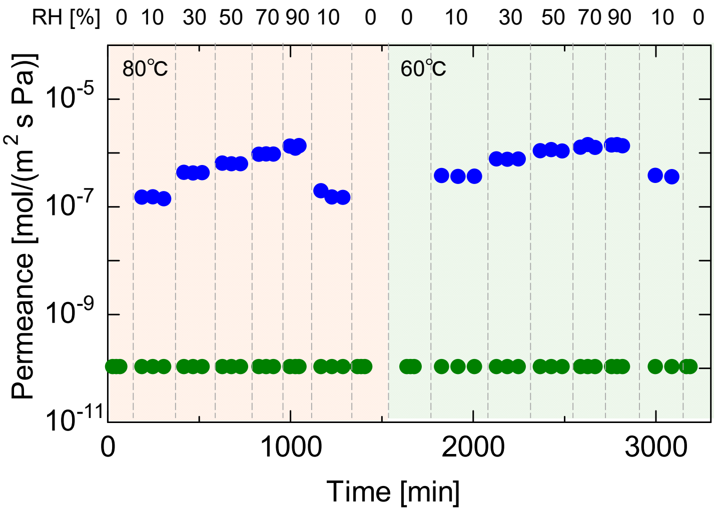

Figure S6. Time course of VP performance at different humidity levels (0 - 90%) through the GO/PES membrane at 60 and 80 °C. (Filtration, GO coating amount: 0.074 mg/cm^2)
